# Transcriptome Analysis of Early Defenses in Rice against *Fusarium fujikuroi*

**DOI:** 10.1186/s12284-020-00426-z

**Published:** 2020-09-10

**Authors:** An-Po Cheng, Szu-Yu Chen, Ming-Hsin Lai, Dong-Hong Wu, Shih-Shun Lin, Chieh-Yi Chen, Chia-Lin Chung

**Affiliations:** 1 Department of Plant Pathology and Microbiology, National Taiwan University, No. 1, Sec. 4, Roosevelt Rd, Taipei City, 10617 Taiwan; 2 Crop Science Division, Taiwan Agricultural Research Institute, No. 189, Zhongzheng Rd., Wufeng Dist, Taichung City, 41362 Taiwan; 3 Department of Agronomy, National Chung Hsing University, No. 145, Xingda Rd., South Dist, Taichung City, 40227 Taiwan; 4 Institute of Biotechnology, National Taiwan University, No. 1, Sec. 4, Roosevelt Rd, Taipei City, 10617 Taiwan

**Keywords:** *Fusarium fujikuroi*, Transcriptome, Jasmonic acid, *OsJAZ*, *XIAO*, *OsWRKY*, *OsERF*

## Abstract

**Background:**

Bakanae is a seedborne disease caused by *Fusarium fujikuroi*. Rice seedlings emerging from infected seeds can show diverse symptoms such as elongated and slender stem and leaves, pale coloring, a large leaf angle, stunted growth and even death. Little is known about rice defense mechanisms at early stages of disease development.

**Results:**

This study focused on investigating early defenses against *F. fujikuroi* in a susceptible cultivar, Zerawchanica karatals (ZK), and a resistant cultivar, Tainung 67 (TNG67). Quantitative PCR revealed that *F. fujikuroi* colonizes the root and stem but not leaf tissues. Illumina sequencing was conducted to analyze the stem transcriptomes of *F. fujikuroi*-inoculated and mock-inoculated ZK and TNG67 plants collected at 7 days post inoculation (dpi). More differentially expressed genes (DEGs) were identified in ZK (*n* = 169) than TNG67 (*n* = 118), and gene ontology terms related to transcription factor activity and phosphorylation were specifically enriched in ZK DEGs. Among the complex phytohormone biosynthesis and signaling pathways, only DEGs involved in the jasmonic acid (JA) signaling pathway were identified. Fourteen DEGs encoding pattern-recognition receptors, transcription factors, and JA signaling pathway components were validated by performing quantitative reverse transcription PCR analysis of individual plants. Significant repression of jasmonate ZIM-domain (*JAZ*) genes (*OsJAZ9*, *OsJAZ10*, and *OsJAZ13*) at 3 dpi and 7 dpi in both cultivars, indicated the activation of JA signaling during early interactions between rice and *F. fujikuroi*. Differential expression was not detected for salicylic acid marker genes encoding phenylalanine ammonia-lyase 1 and non-expressor of pathogenesis-related genes 1. Moreover, while MeJA did not affect the viability of *F. fujikuroi*, MeJA treatment of rice seeds (prior to or after inoculation) alleviated and delayed bakanae disease development in susceptible ZK.

**Conclusions:**

Different from previous transcriptome studies, which analyzed the leaves of infected plants, this study provides insights into defense-related gene expression patterns in *F. fujikuroi*–colonized rice stem tissues. Twelve out of the 14 selected DEGs were for the first time shown to be associated with disease resistance, and JA-mediated resistance was identified as a crucial component of rice defense against *F. fujikuroi*. Detailed mechanisms underlying the JA-mediated bakanae resistance and the novel defense-related DEGs are worthy of further investigation.

**Supplementary information:**

**Supplementary information** accompanies this paper at 10.1186/s12284-020-00426-z.

## Background

Bakanae disease, caused by the heterothallic ascomycete fungus *Fusarium fujikuroi* Nirenberg, has become a threat to rice quality and yield in recent years. Many studies from Asian countries such as Bangladesh, India, South Korea, Pakistan, and Taiwan have reported the increasing incidence and severity of bakanae disease (Khan et al. 2000; Chu et al. 2010; Haq et al. 2010; Gupta et al. 2015; Kim et al. 2015). *F. fujikuroi* is a seed-borne pathogen that can infect rice panicles at the flowering stage (Ou 1985). Rice seedlings emerging from infected seeds can show diverse symptoms such as elongated and slender stem and leaves, pale coloring, a large leaf angle, stunted growth and even death. The conventional disease management strategy for bakanae disease is seed disinfection using fungicides. However, *F. fujikuroi* isolates resistant to benzimidazole, prochloraz, or tebuconazole have been reported (Chen et al. 2014; Kim et al. 2010; Chen et al. 2016). Because bakanae disease is becoming a serious threat to rice production, it is crucial to develop new control measures from different perspectives.

Bakanae resistance has been explored by large-scale screening of rice germplasm and quantitative trait locus (QTL) mapping. Research groups in India (Fiyaz et al. 2014), Korea (Kim et al. 2014), and Taiwan (Chen et al. 2019) used high-throughput inoculation methods to screen for resistant materials from 92, 500, and 231 diverse rice varieties, respectively. A total of 28 QTLs for bakanae resistance were mapped on rice chromosomes 1, 3, 4, 6, 8, 9, 10 and 11 (Volante et al. 2017; Fiyaz et al. 2016; Lee et al. 2018; Cheon et al. 2019; Ji et al. 2018a; Hur et al. 2015; Lee et al. 2019; Kang et al. 2019; Yang et al. 2006; Chen et al. 2019). Chromosome 1, where 13 QTLs are located, appears to be particularly important. Using four different bi-parental populations and rice diversity panel 1 (RDP1) (Cheon et al. 2019; Ji et al. 2018a; Hur et al. 2015; Lee et al. 2019; Fiyaz et al. 2016; Chen et al. 2019), co-localized QTLs were fine-mapped to a region (21.36–24.37 Mb on chromosome 1) crucial for bakanae resistance. Although QTLs have been associated with mortality rate, disease incidence, disease severity, and *F. fujikuroi* colonization, no causal genes have been cloned and functionally characterized.

Rice responses to *F. fujikuroi* have been investigated in two transcriptome analyses (Ji et al. 2016; Matić et al. 2016). Ji et al. (2016) examined the RNA from the leaves of a moderately resistant cultivar, 93–11, and a susceptible cultivar, Nipponbare, at 7 days after treatment. Upon infection by *F. fujikuroi*, three *WRKYs* (*OsWRKY107*, *OsWRKY13* and *OsWRKY71*), a *wall-associated kinase* (*OsWAK112d*), and two *mitogen-activated protein kinase kinase kinase* (*MAP3K.4* and *MAP3K.5*) genes were up-regulated in resistant 93–11, and five *pollen Ole e I* (*POEI*) genes (*POEI11* to *POEI15*), which are known to be involved in the response to abiotic stress, were greatly induced in susceptible Nipponbare. It was also found that gene ontology (GO) terms related to reactive oxygen species generation and detoxification were enriched in both 93–11 and Nipponbare. Using another resistant cultivar, Selenio, and susceptible cultivar, Dorella, Matić et al. (2016) studied the transcriptomes of rice leaves at 7 and 21 days post inoculation (dpi). In general, *F. fujikuroi* infection induced the expression of *glycoside hydrolases*, *MAPKs*, and *WRKYs* in resistant Selenio, but caused up-regulation of *chitinases* and down-regulation of *MAPKs* and *WRKYs* in susceptible Dorella at 21 dpi.

Phytohormones and phytoalexins have been implicated in bakanae resistance. In the study by Matić et al. (2016), the GO term “jasmonic acid biosynthetic process” was specifically enriched in genes differentially expressed upon *F. fujikuroi* infection in resistant cultivar Selenio, whereas the GO terms “response to salicylic acid stimulus” and “gibberellin metabolic process” were specifically enriched in genes differentially expressed in susceptible Dorella at 21 dpi. By quantifying phytohormones and phytoalexins in rice leaves and culms using HPLC−MS/MS, Siciliano et al. (2015) found that inoculation of *F. fujikuroi* induced the accumulation of gibberellin (GA) and abscisic acid (ABA) and reduced the amount of jasmonic acid (JA) in susceptible Dorella at 3 and 4 weeks after seed germination. Enhanced levels of phytoalexins, mainly sakuranetin, were detected in *F. fujikuroi*-inoculated resistant cultivar Selenio.

While Ji et al. (2016) and Matić et al. (2016) have revealed abundant genes differentially expressed in the leaves of *F. fujikuroi*-inoculated plants, their sampling strategies may have led to the discovery of genes involved in systemic rather than localized defensive responses. According to our previous evaluation of *F. fujikuroi* colonization on eight rice cultivars at 21 dpi, the average re-isolation frequencies of *F. fujikuroi* were 96%, 67%, 40%, 32%, and 25% from the stem segments 0–1 cm, 1–2 cm, 2–3 cm, 3–4 cm, and 4–5 cm above the base of the infected seedlings, respectively (Chen et al. 2015). This suggested a localized but not systemic infection of *F. fujikuroi* at early stage of disease development.

In this study, to understand the early lines of defense against *F. fujikuroi* in a susceptible cultivar, Zerawchanica karatals (ZK), and a resistant cultivar, Tainung 67 (TNG67), we first clarified the initial extent of *F. fujikuroi* colonization in different rice tissues by performing real-time quantitative PCR (qPCR) assays. We found that the stem serves as an initial colonization site so the stem tissues at 7 dpi were selected as a target for transcriptome analysis. A set of genes up- and down-regulated in response to *F. fujikuroi* infection, including genes involved in signal perception and transduction plant defense pathways, were identified and validated by real-time quantitative reverse transcription PCR (qRT-PCR) of individual plant samples from additional independent trials. To verify the involvement of JA signaling in rice resistance against bakanae disease, the effects of exogenous JA on rice and *F. fujikuroi* were also examined.

## Materials and Methods

### Plant Materials

A susceptible rice cultivar, ZK, and a resistant rice cultivar, TNG67, were used in this study. These cultivars were selected based on resistance screening of 231 diverse rice accessions from Rice Diversity Panel 1 (RDP1) (Chen et al. 2019). ZK was moderately susceptible and TNG67 was moderately resistant (Additional file 8: Fig. S1). In repeated experiments, the two cultivars grew well and showed consistent symptoms after *F. fujikuroi* inoculation under growth chamber conditions. Rice seeds were provided by the Genetics Stocks Oryza (GSOR) germplasm collection (Agricultural Research Service, US Department of Agriculture) and multiplied in the field at the Crop Science Division, Taiwan Agricultural Research Institute.

### Inoculation of *F. fujikuroi*

*F. fujikuroi* isolate Ff266 was cultured on 1/2 potato dextrose agar (PDA) for 4 days at 25 °C under a 12-h photoperiod. The conidia were collected using sterile dH_2_O and a sterile tip, then adjusted to 1 × 10^5^ spores/ml. Rice seeds were surface sterilized in 60 °C sterile dH_2_O for 10 min then immersed in sterile dH_2_O for 2 days at room temperature. The pre-germinated seeds were soaked in the spore suspension (inoculated) or sterile dH_2_O (mock) overnight, then sown in pots (L x W x H = 3.5 × 4.5 × 5.5 cm) filled with Akadama soil (a granular volcanic clay-like mineral naturally occurring in Japan). Plants were cultivated in the dark for the first 2 days and subsequently under a 12-h photoperiod in a walk-in chamber set at a 32 °C day/28 °C night temperature. Disease severity index (DSI) was calculated based on visual ratings of individual plants at 21 dpi using a 0–3 scale (Chen et al. 2016; Chen et al. 2019) as follows: $\mathrm{DSI}=\frac{\sum \mathrm{Rating}\ \mathrm{scale}\times \mathrm{No}.\mathrm{of}\ \mathrm{seedlings}\ \mathrm{at}\ \mathrm{the}\ \mathrm{scale}}{\operatorname{Max}.\mathrm{scale}\times \mathrm{Total}\ \mathrm{No}.\mathrm{of}\ \mathrm{seedlings}}\times 100\%$.

### DNA Extraction and Quantification of *F. fujikuroi* in Different Rice Tissues

qPCR was conducted to determine the sites colonized by *F. fujikuroi* in rice. Inoculation of ZK and TNG67 with *F. fujikuroi* Ff266 was conducted as described above in two independent trials, with 6–8 plants per cultivar in each trial. The aerial and root samples were collected at 3 dpi, and the leaf, stem, and root samples were collected at 7 dpi. In this study, the stem was defined as the aerial part between the base and the second node of a seedling (Fig. 2 in the study of Chung et al. 2016). DNA extraction and qPCR analysis were conducted on an individual plant basis. Genomic DNA was extracted from different tissues of the inoculated and non-inoculated healthy rice plants, and from 4-day-old *F. fujikuroi* colonies cultured on 1/2 PDA using a standard cetyltrimethylammonium bromide (CTAB) extraction method (Doyle and Doyle 1987). DNA concentration was measured with a NanoDrop® ND-1000 spectrophotometer (Thermo Scientific, Wilmington, DE, USA). qPCR was performed in three technical replicates with the ABI Prism 7500 sequence detection system (Applied Biosystems, Carlsbad, CA, USA). Each qPCR reaction contained 5 μl SYBR Premix EX Taq II (Ti RNase H Plus) (Takara Bio, Shiga, Japan), 0.5 μl of 10 μM forward primer, 0.5 μl of 10 μM reverse primer, 1 μl (100 ng) DNA, and 3 μl ddH_2_O. The primers TqF2 (5′-GGCGCGTTTTGCCCTTTCCT-3′) and TqR (5′-AGCGGCTTCCTATTGTCGAA-3′) (Carneiro et al. 2017) specifically targeting the *translation elongation factor 1-α* gene in *F. fujikuroi* were used. Standard curves were generated for different rice tissues by mixing a serial dilution of *F. fujikuroi* DNA (10 ng, 2 ng, 400 pg, 80 pg, and 16 pg) and healthy rice DNA (90 ng, 98 ng, 100 ng, 100 ng, and 100 ng of DNA from different tissues of ZK and TNG67).

### RNA Extraction and cDNA Preparation

Total RNA was extracted using TRIzol® Reagent (Invitrogen™ Life Technology, Carlsbad, CA, USA) following the manufacturer’s instructions. The TURBO DNA-free™ Kit (Invitrogen™ Life Technology, Carlsbad, CA, USA) was used to remove potentially contaminating DNA. RNA concentration was measured using a NanoDrop® ND-1000 spectrophotometer (Thermo Scientific, Wilmington, DE, USA). cDNA was synthesized using the PrimeScript™ RT Reagent Kit (Takara Bio, Shiga, Japan) following the manufacturer’s instructions.

### Transcriptome Analysis

Total RNA samples were extracted from the stem tissues of *F. fujikuroi*-inoculated and dH_2_O-treated ZK and TNG67 seedlings collected at 7 dpi from two independent inoculation trials (carried out in August and December, 2015). The stem tissues were cut with scissors and snap-frozen in liquid nitrogen. Each RNA sample was extracted from stem tissues pooled from 40 individual plants per cultivar per treatment per trial. In each trial, to ensure that the inoculation was successful, we kept another set of plants for continuous observation of symptom development until 21 dpi. Eight RNA samples [two cultivars, two treatments (inoculated and mock), and two biological replicates (each containing 40 plants from an independent inoculation trail)] were used for strand-specific RNA sequencing (insert size 150–180 bp; 2 × 150 bp paired-end reads) on the Illumina HiSeq® 2500 Sequencing System at Sequencing Technology Company Limited (Taipei, Taiwan). The quality of RNA and the libraries were inspected using an Agilent 2100 bioanalyzer (Agilent Technologies, Palo Alto, CA, USA).

Transcriptome data were analyzed using the ContigViews web server (www.contigviews.bioagri.ntu.edu.tw) (Liu et al. 2014). Bowtie 2 (Langmead and Salzberg 2012) was used for calculation of gene expression and reference based mapping (default parameters). Rice Os-Nipponbare-Reference-IRGSP-1.0 [from Ensembl Genomes (Kersey et al. 2017)] was used as a reference for read mapping and gene annotation. RNA-seq data were deposited in the NCBI Sequence Read Archive database under the accession numbers SAMN13972374 to SAMN13972381.

Principal component analysis (PCA) was conducted to determine the relatedness of different samples. The PCA plot was generated using the DESeq package version 1.36.0 in R with the plotPCA function (Anders and Huber 2010). For each cultivar, differential gene expression between *F. fujikuroi*-inoculated and control seedlings was analyzed using the DESeq package version 1.36.0 in R with the “pooled-CR” method and a “maximum” sharing mode (Anders and Huber 2010). Genes with *p* value ≦ 0.05, log_2_ fold change (log_2_FC) ≧ |1|, and Fragments Per Kilobase of transcript per Million mapped reads (FPKM) ≧ 1 were recognized as significantly differentially expressed genes (DEGs). DEGs were subject to GO term enrichment analysis [false discovery rate (FDR) (Hochberg) ≦ 0.1] using AgriGO v.2 (Du et al. 2010; Tian et al. 2017). Mapman 3.6.0 (Thimm et al. 2004) was used to analyze the DEGs in the biotic stress and pattern-recognition receptor (PRR) categories. DEGs were also input into Reactome (Croft et al. 2013; Fabregat et al. 2017) for molecular pathway analysis. Plant Transcription Factor Database v4.0 (PlantTFDB) (Jin et al. 2017; Jin et al. 2015; Jin et al. 2013) was used for prediction and classification of transcription factors (TFs).

### qRT-PCR Analysis

To validate the expression of candidate genes, an additional two independent inoculation trials were conducted. The stem tissues were collected at 3 dpi and 7 dpi from six to eight individual plants per cultivar per treatment per trial. RNA extraction and cDNA synthesis were conducted as described above. Relative gene expression was measured (on an individual plant basis) in three technical replicates with the ABI Prism 7500 sequence detection system (Applied Biosystems, Carlsbad, CA, USA). Each qRT-PCR reaction contained 5 μl SYBR Premix EX Taq II (Ti RNase H Plus) (Takara Bio, Shiga, Japan), 0.5 μl of 10 μM forward primer, 0.5 μl of 10 μM reverse primer, 1 μl cDNA, and 3 μl ddH_2_O. Primer sequences for qRT-PCR are listed in Additional file 1: Table S1 (Jain et al. 2017; Li et al. 2016; Sathe et al. 2019; Manosalva et al. 2009; Zhang et al. 2009). Primers were designed using Primer 3 Plus (Version 2.4.1) (Untergasser et al. 2012). To avoid nonspecific binding, candidate primers were further analyzed using prfectBLAST (Santiago-Sotelo and Ramirez-Prado 2012) to perform searches against the rice genome. qRT-PCR was performed using the following thermal cycling parameters: 95 °C for 30 s followed by 40 cycles of 5 s at 95 °C and 34 s at 60 °C. The rice gene *Elongation factor 1-alpha* (*OsEF1α*) was used as an internal control for normalization of the cycle threshold (Ct) values in different samples (Manosalva et al. 2009). The relative gene expression levels were calculated by the comparative Ct (2^-△△Ct^) method (Livak and Schmittgen 2001).

### MeJA Treatment

Rice seeds were soaked in 0.1 mM or 0.01 mM methyl jasmonate (MeJA) (Sigma–Aldrich, St. Louis, MO, USA) or ddH_2_O (control) for 8 h at 25 °C before or after *F. fujikuroi* inoculation. The MeJA solutions and ddH_2_O were adjusted to pH = 5. The concentrations of MeJA were chosen according to previous studies on the effects of exogenous JA on disease resistance in rice (Ji et al. 2015; Chen et al. 2018). The inoculation of *F. fujikuroi* and rating of disease severity at 14 dpi and 21 dpi were conducted as described above. The experiment was performed in two independent trials, each with 12–15 individual plants per treatment per cultivar.

The effects of MeJA on *F. fujikuroi* were also assessed. For the spore germination test, 10 μl of *F. fujikuroi* spore suspension (5 × 10^4^ spores/ml in 1/2 potato dextrose broth) mixed with an equal volume of MeJA (final concentration: 0.1 mM or 0.01 mM) or ddH_2_O (pH adjusted to 5) was placed on a glass slide. After incubation in a moist chamber for 12 h at 25 °C, 100 spores on each glass slide were inspected for germination under the microscope. A spore with a protruding germ tube two times longer than its largest diameter was considered germinated (Chen et al. 2016). The experiment was performed in two independent trials, each with three glass slides per treatment. For the colony growth test, 100 μl of *F. fujikuroi* spores (1 × 10^5^ spores/ml in ddH_2_O) was mixed with an equal volume of MeJA (final concentration: 0.1 mM or 0.01 mM) or ddH_2_O (pH adjusted to 5). After incubation for 8 h at 25 °C, 10 μl of the spore suspension was transferred to a 1/2 PDA plate. Colony diameters were measured after 7 and 10 days of incubation at 25 °C under a 12-h photoperiod. The experiment was performed in two independent trials, each with three to five plates per treatment.

### Statistical Analysis

A two-tailed unpaired Student’s *t*-test was conducted to analyze the differences between the DSIs of ZK and TNG67 at *p* < 0.05. Differences among multiple treatments were analyzed by one-way analysis of variance (ANOVA) with Tukey’s multiple comparison test at *p* < 0.05. Statistical analyses and graphing were performed using GraphPad Prism version 7.04 (GraphPad Software, La Jolla California USA).

## Results

### Disease Symptoms of *F. fujikuroi*-Inoculated ZK and TNG67

**Fig. 1:**
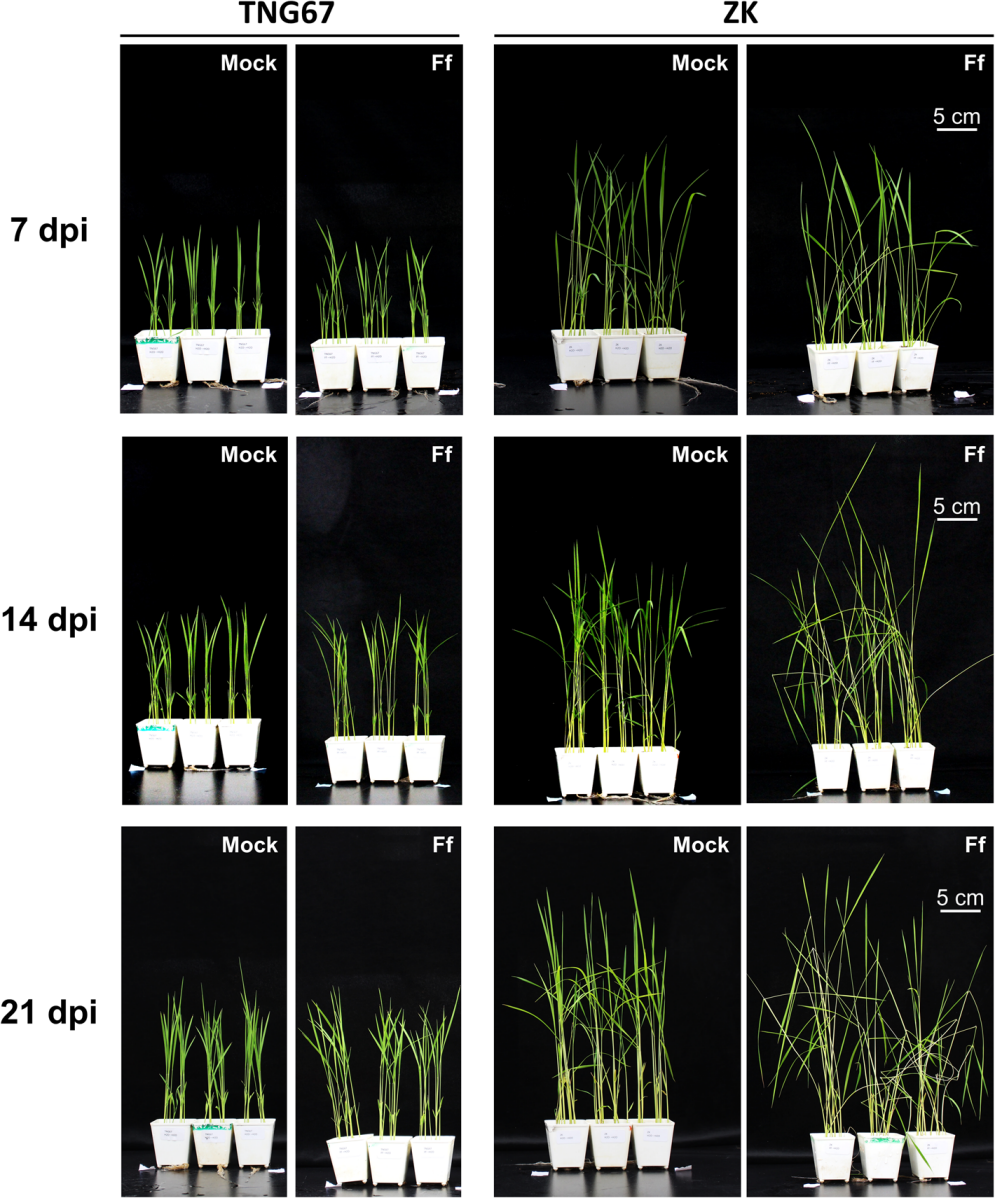
Symptoms of Zerawchanica karatals (ZK) and Tainung 67 (TNG67) after dH_2_O (mock) or *Fusarium fujikuroi* (Ff) inoculation at 7, 14, and 21 days post inoculation (dpi)

The symptoms of ZK and TNG67 inoculated with *F. fujikuroi* Ff266 are shown in Fig. 1 and Additional file 9: Fig. S2. ZK started to exhibit mild symptoms at 7–10 dpi and showed typical bakanae disease symptoms such as abnormal stem elongation, a slender stem, and a large leaf angle after 14 dpi. On the contrary, TNG67 showed no or only one type of bakanae symptoms at 7, 14, and 21 dpi. The DSIs at 21 dpi were 56.2 ± 3.4% (mean ± SE) for ZK and 11.0 ± 1.9% for TNG67 (*p* < 0.0001). To clarify the early responses of the two cultivars before full development of disease symptoms, we chose 7 dpi as the time point for subsequent transcriptome analysis.

### Quantification of *F. fujikuroi* in Different Rice Tissues

**Fig. 2:**
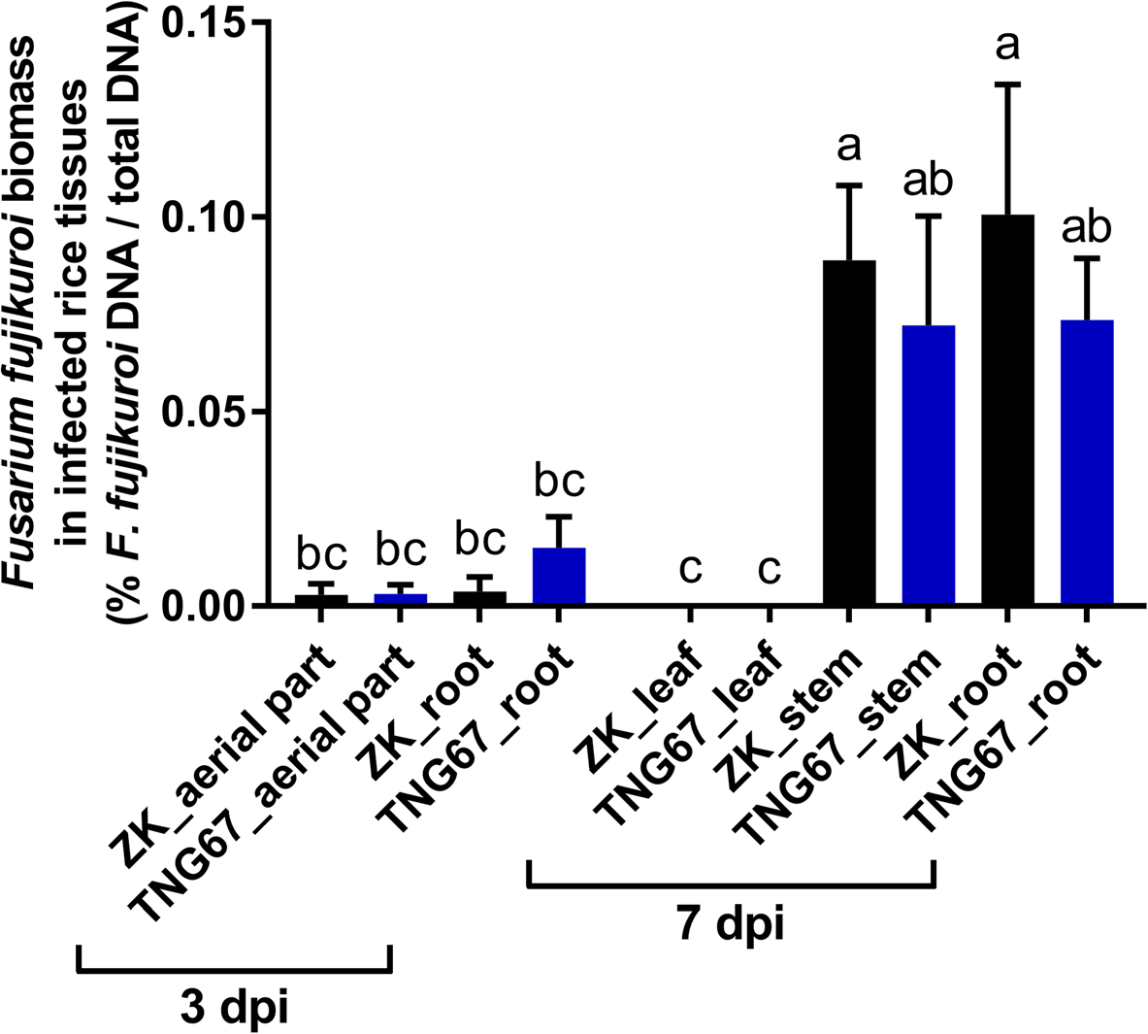
Quantification of *Fusarium fujikuroi* in different tissues of Zerawchanica karatals (ZK) and Tainung 67 (TNG67) at 3 and 7 days post inoculation (dpi). Data are mean ± SEM (*n* = 2 independent trials with six to eight plants per treatment per trial). Different letters indicate significant difference based on Tukey’s multiple comparison test at *p* < 0.05

Focusing on the early stages of disease development, qPCR was conducted to determine the levels of *F. fujikuroi* colonization in different parts of rice seedlings at 3 and 7 dpi. In our qPCR assays, the coefficient of determination (*R*^2^) values for the standard curves based on different rice tissues were all > 0.99 (Additional file 10: Fig. S3). Similar amounts of *F. fujikuroi* were detected in the roots and aerial parts of both cultivars at 3 dpi. For both cultivars at 7 dpi, *F. fujikuroi* colonization was detected in the root and stem but not in the leaf tissues (Fig. 2). No significant differences were detected between the root and stem tissues or between the two cultivars.

### Transcriptome Profiling and Identification of Differentially Expressed Genes

RNA sequencing was conducted to reveal gene expression patterns in the stem tissues of ZK and TNG67 at 7 dpi. The number of reads and mapping rates for the eight RNA libraries are shown in Additional file 2: Table S2. An average of 7.5 Gb raw reads (40–62 million sequences, 150 bp in length) was generated for each sample, and 79.13%–82.95% of clean reads were mapped to the reference exon regions. The total numbers of transcripts identified were 35,683 in ZK and 35,926 in TNG67. The expression levels of all transcripts are provided in Additional file 3: Table S3. PCA was conducted to assess transcriptional variation among cultivars, treatments, and trials (Additional file 11: Fig. S4a). A distinct separation between ZK and TNG67 was observed along PC1. PC2 showed that the same trial-cultivar combination clustered more closely together, suggesting that the variability between treatments were smaller than between different trials.

By comparing *F. fujikuroi*-inoculated libraries with the mock libraries, 169 and 118 DEGs were identified in ZK and TNG67 (Additional file 11: Fig. S4b and Additional file 4: Table S4), respectively. The expression profiles of the DEGs in ZK and TNG67 are shown in Additional file 11: Fig. S4c. There were 40 up-regulated DEGs in ZK and 87 in TNG67, and 129 down-regulated DEGs in ZK and 31 in TNG67. ZK and TNG67 only shared two DEGs: *OsWRKY71* (Os02g0181300; down-regulated in both cultivars) and *OsDBH* (DEAD-Box Helicase, Os04g0486800; down-regulated in ZK and up-regulated in TNG67).

### GO Enrichment Analysis

**Fig. 3:**
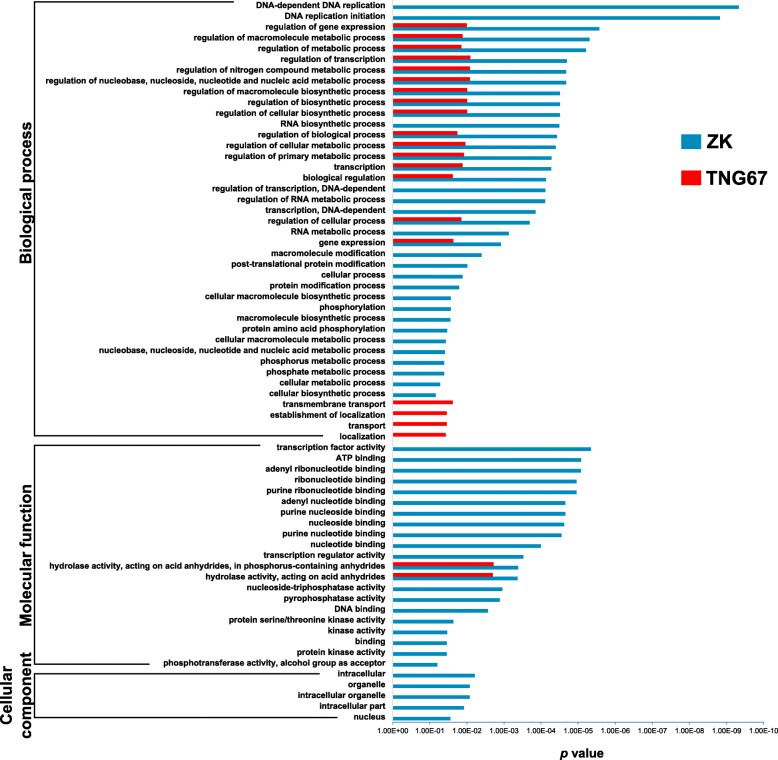
Gene ontology (GO) enrichment analyses of the genes differentially expressed in Zerawchanica karatals (ZK) and Tainung 67 (TNG67) after the inoculation of *Fusarium fujikuroi*. Enriched GO terms were filtered using an FDR cutoff of 0.1. X-axis indicates the enrichment *p* values. Blue bars: Zerawchanica karatals (ZK); red bars: Tainung 67 (TNG67)

DEGs were classified into three major GO categories: biological process, molecular function, and cellular component. For ZK and TNG67, 63 and 22 enriched GO terms were identified, respectively (Fig. 3). TNG67 showed no enriched GO terms in the cellular component category. Moreover, most of the GO terms enriched in TNG67 DEGs [all terms except for “transmembrane transport” (GO:0055085), “transport” (GO:0006810), “establishment of localization” (GO:0051234), and “localization” (GO:0051179)] were also enriched in ZK DEGs. Some GO terms were specific to ZK, including “transcription factor activity” (GO:0003700), “post-translational protein modification” (GO:0043687), and four GO terms associated with phosphorylation [i.e., “phosphorus metabolic process” (GO:0006793), “phosphate metabolic process” (GO:0006796), “phosphorylation” (GO:0016310), and “protein amino acid phosphorylation” (GO:0006468)]. GO terms associated with plant responses to biotic stresses were not enriched in TNG67 or ZK DEGs.

### DEGs Related to Biotic Stress and Pattern Recognition Receptors

Mapman analysis identified 57 DEGs (37 in ZK and 21 in TNG67, with 1 DEG in both cultivars) in the biotic stress category (Additional file 4: Table S4). These included 9 PRRs and 17 TFs. While a large proportion of biotic stress-related genes were down-regulated in susceptible ZK, more up-regulated genes were found in resistant TNG67 (ZK: 31 down- and 6 up-regulated biotic stress DEGs; TNG67: 12 up- and 9 down-regulated biotic stress DEGs).

Plants deploy membrane-associated receptor-like kinases and receptor-like proteins as PRRs to detect a wide range of pathogen- or damage-associated molecular patterns (PAMPs or DAMPs). PAMP-PRR pairs function as multi-protein complexes to activate defense signaling pathways and responses, known as pattern-triggered immunity (Monaghan and Zipfel 2012; Zipfel 2014). This study identified 10 differentially expressed PRRs, six in ZK [Os03g0297800, Os04g0576900 (*XIAO*), Os05g0207700, Os07g0628700, Os08g0117700, Os08g0501700 (*OsWAK76*)] and four in TNG67 [Os06g0134700, Os07g0550900, Os09g0110100, Os12g0145900] (Additional file 4: Table S4). Notably, in ZK, all except *OsWAK76* were down-regulated; but in TNG67, all except *Os07g0550900* were up-regulated.

### DEGs Annotated as TFs

TFs regulate the transcription of target genes by binding to specific DNA regions (Latchman 1997). TFs and their transcriptional regulatory networks play crucial roles in plant development and stress responses. Twenty-three out of 169 (13.6%) DEGs in ZK and 14 out of 118 (11.9%) DEGs in TNG67 were annotated as TFs (Additional file 4: Table S4). These included five WRKYs and seven ethylene response factors (ERFs). WRKY proteins bind to W-box elements in the promoter regions of many defense-related genes (Chen and Ronald 2011; Dong et al. 2003). The five differentially expressed WRKYs [*OsWRKY21* (Os01g0821600) and *OsWRKY24* (Os01g0826400) in ZK; *OsWRKY1* (Os01g0246700) and *OsWRKY28* (Os06g0649000) in TNG67, and *OsWRKY71* (Os02g0181300) in both ZK and TNG67] were all down-regulated. ERFs have been reported to be involved in biotic and abiotic responses, hormone signaling transduction, and development (Nakano et al. 2006). ERFs bind to promoter regions containing AGCCGCC motifs (GCC box) to trigger stress-responsive gene expression (Müller and Munné-Bosch 2015; Ku et al. 2018). Seven down-regulated ERFs [*OsERF53* (Os01g0224100), *OsERF54* (Os01g0657400), *OsERF25* (Os02g0677300), *OsERF30* (Os04g0572400), *OsERF26* (Os06g0127100), *OsERF104* (Os08g0474000), *OsERF133* (Os09g0522100)] were identified in ZK. None of the ERFs in resistant TNG67 were identified as DEGs.

### DEGs Involved in the JA Signaling Pathway

The expression of genes involved in JA, ethylene, salicylic acid (SA), and GA biosynthesis and signaling pathways are shown in Additional file 5: Table S5. Differential expression was not observed for all genes participating in biosynthesis pathways. Among the genes involved in various phytohormone signaling pathways, only four associated with JA signaling were identified as DEGs in ZK: three down-regulated jasmonate ZIM-domain (*JAZ*) genes [*OsJAZ9* (Os03g0180800), *OsJAZ10* (Os03g0181100), and *OsJAZ13* (Os10g0391400)] and an up-regulated histone deacetylase (*HDA*) gene, *HDA703* (Os02g0214900; log_2_FC = 3.02). JAZ proteins can directly bind to the basic helix-loop-helix TFs MYCs, resulting in inhibition of the expression of JA-responsive genes (Cheng et al. 2011; Fernández-Calvo et al. 2011). HDAs can be recruited by the transcription co-repressor TOPLESS, which leads to chromatin remodeling and suppression of JA-responsive gene expression (Long et al. 2006; Wu et al. 2008).

### qRT-PCR Analysis

**Fig. 4:**
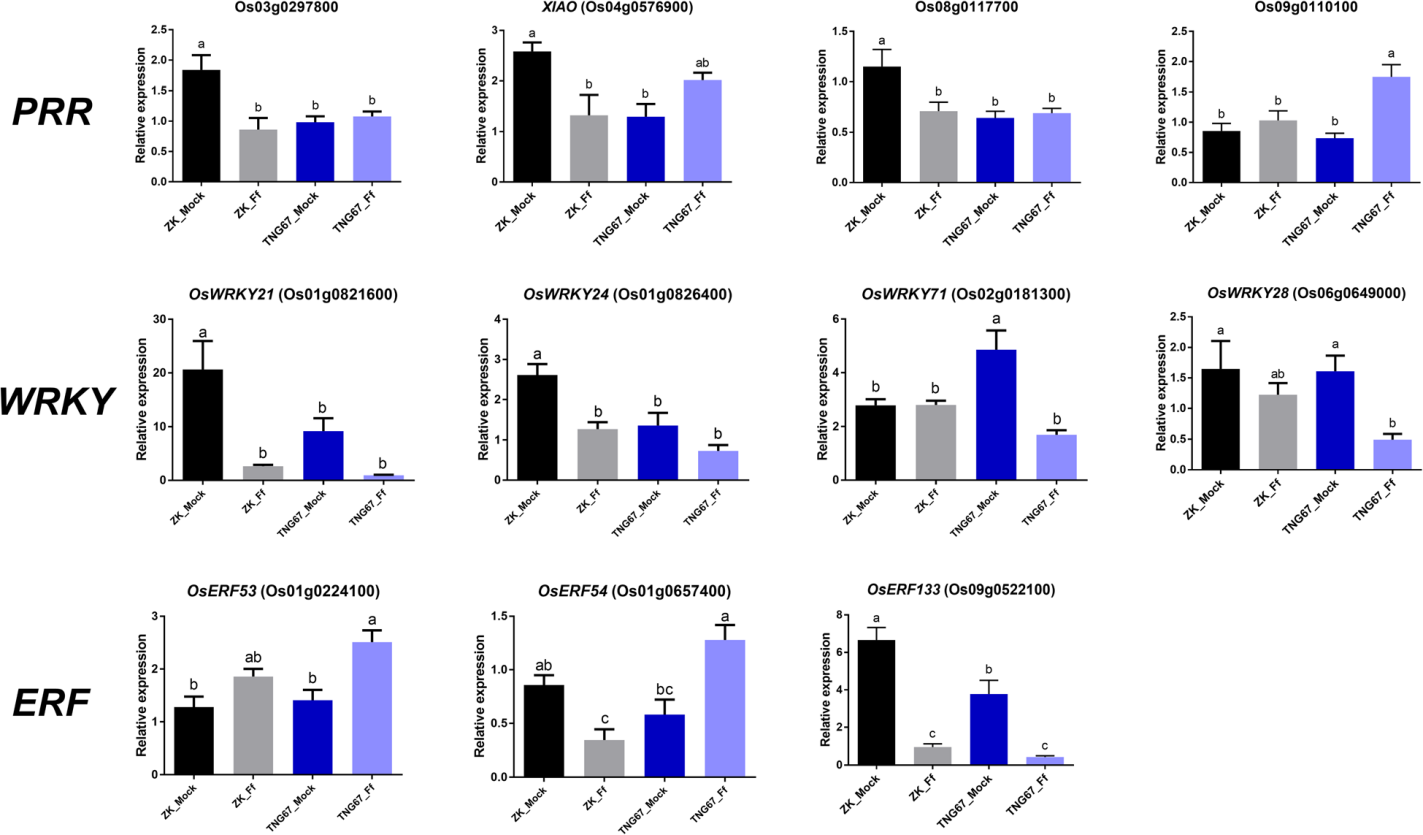
Expression profiling of pattern-recognition receptor (*PRR*), *WRKY*, and ethylene response factor (*ERF*) genes in Zerawchanica karatals (ZK) and Tainung 67 (TNG67) after dH_2_O (mock) or *Fusarium fujikuroi* (Ff) inoculation by real-time quantitative RT-PCR. Samples were collected at 7 days post inoculation. The relative expression level is expressed as the fold change compared with the internal control (*OsEF1α*). Data are mean ± SEM (*n* = six to eight plants per treatment). Different letters indicate significant difference based on Tukey’s multiple comparison test at *p* < 0.05

**Fig. 5:**
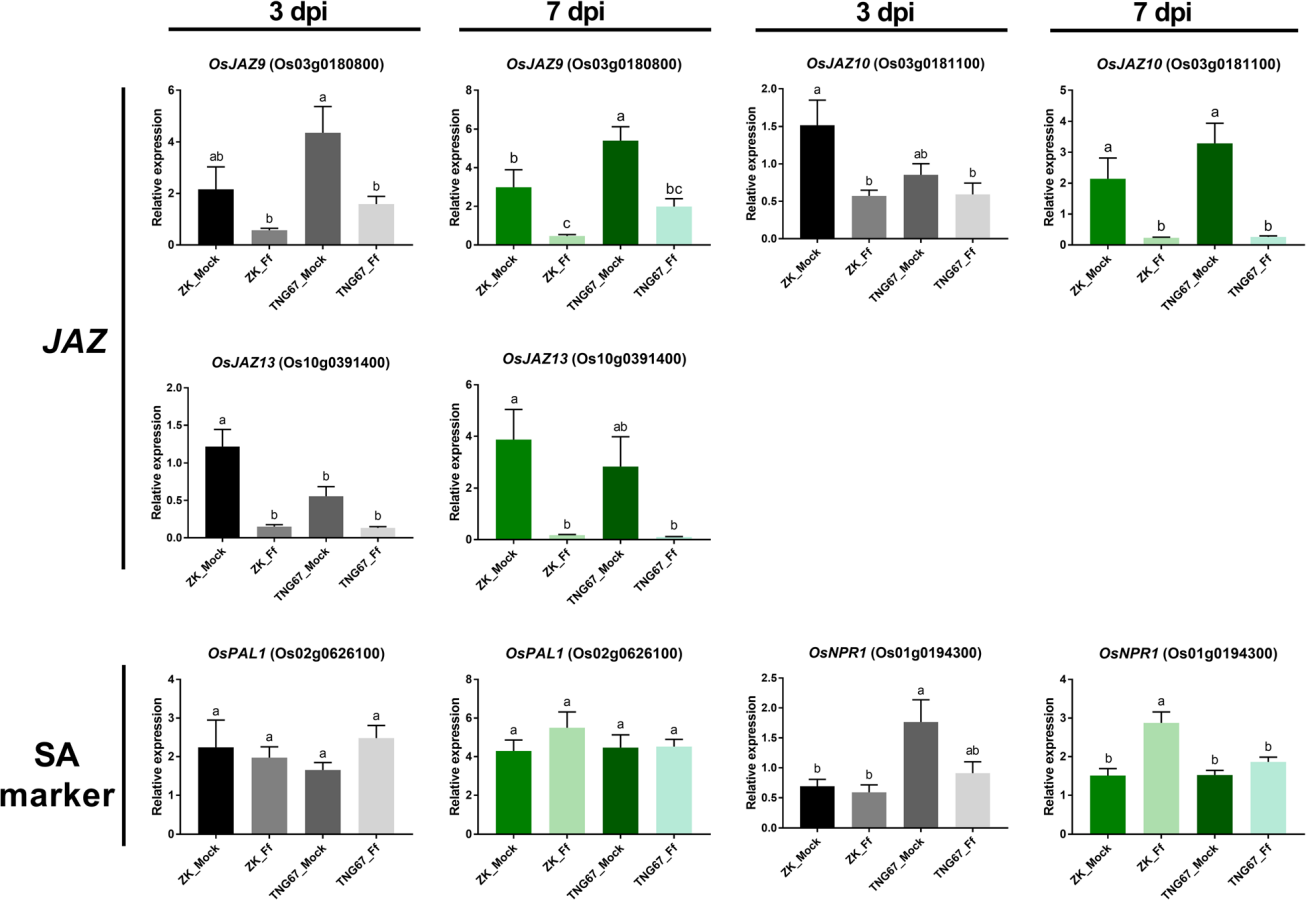
Expression profiling of jasmonate ZIM-domain (*JAZ*) genes and salicylic acid (SA) marker genes in Zerawchanica karatals (ZK) and Tainung 67 (TNG67) after dH_2_O (mock) or *Fusarium fujikuroi* (Ff) inoculation by real-time quantitative RT-PCR. Samples were collected at 3 and 7 days post inoculation (dpi). The relative expression level is expressed as the fold change compared with the internal control (*OsEF1α*). Data are mean ± SEM (*n* = six to eight plants per treatment). Different letters indicate significant difference based on Tukey’s multiple comparison test at *p* < 0.05

qRT-PCR was conducted to quantify the expression levels of 11 genes at 7 dpi and 5 genes at 3 dpi and 7 dpi (Fig. 4 and Fig. 5 show the results from one of two independent trials in which similar trends were observed; log_2_FC values from two trials are in Additional file 6: Table S6). These genes were chosen because of their potential functions in disease resistance and their significant induction or repression in response to *F. fujikuroi*. They included four PRRs (Os03g0297800, *XIAO*, Os08g0117700, Os09g0110100), four *WRKYs* (*OsWRKY21*, *OsWRKY24*, *OsWRKY28*, and *OsWRKY71*), three *ERFs* (*OsERF53*, *OsERF54*, and *OsERF133*), three *JAZs* (*OsJAZ9*, *OsJAZ10*, and *OsJAZ13*), and two SA marker genes [phenylalanine ammonia-lyase (*OsPAL1*, Os02g0626100) and non-expressor of pathogenesis-related (*OsNPR1*, Os01g0194300)]. All of the selected genes except for the two SA marker genes are DEGs identified from the transcriptome analysis. All 14 selected DEGs were confirmed to be significantly up- or down-regulated in *F. fujikuroi*-inoculated ZK and/or TNG67 (Fig. 4 and Additional file 6: Table S6). The qRT-PCR results were generally consistent with the transcriptome data. The only exceptions were *OsWRKY71* and *OsERF53* in ZK (they were found to be down-regulated at 7 dpi in ZK according to the transcriptome analysis; however, no significant difference was detected by qRT-PCR; on the other hand, the down-regulation of *OsWRKY71* and up-regulation of *OsERF53* in TNG67 were confirmed by qRT-PCR).

Five genes functioning in JA- and SA-related pathways were tested for their expression at 7 dpi and an earlier stage (3 dpi) (Fig. 5 and Additional file 6: Table S6). The JA signaling pathway genes *OsJAZ9*, *OsJAZ10*, and *OsJAZ13* were significantly down-regulated at 7 dpi in ZK and TNG67 (log_2_FC = − 1.44 to − 5.69). Down-regulation was also observed at 3 dpi, but to a smaller extent (log_2_FC = − 0.53 to − 3.59). At 3 dpi, significant down-regulation of *OsJAZ9* in TNG67 and *OsJAZ10* and *OsJAZ13* in ZK was observed; a slight decrease (not significant) was observed for *OsJAZ9* in ZK and *OsJAZ10* and *OsJAZ13* in TNG67. For *OsPAL1*, which encodes a key enzyme that catalyzes the biosynthesis of SA and phenolic compounds (Lee et al. 1995; D'Maris Amick Dempsey et al. 2011), no differential expression was found between the inoculated and mock samples at 3 and 7 dpi in both cultivars. For *OsNPR1*, which encodes a positive regulator controlling SA-mediated defense responses and systemic acquired resistance (Yuan et al. 2007; Dong 2004), we detected no significant difference at 3 dpi in both cultivars, but significant up-regulation at 7 dpi in ZK (log_2_FC = 0.93).

### MeJA-Induced Resistance to *F. fujikuroi*

**Fig. 6:**
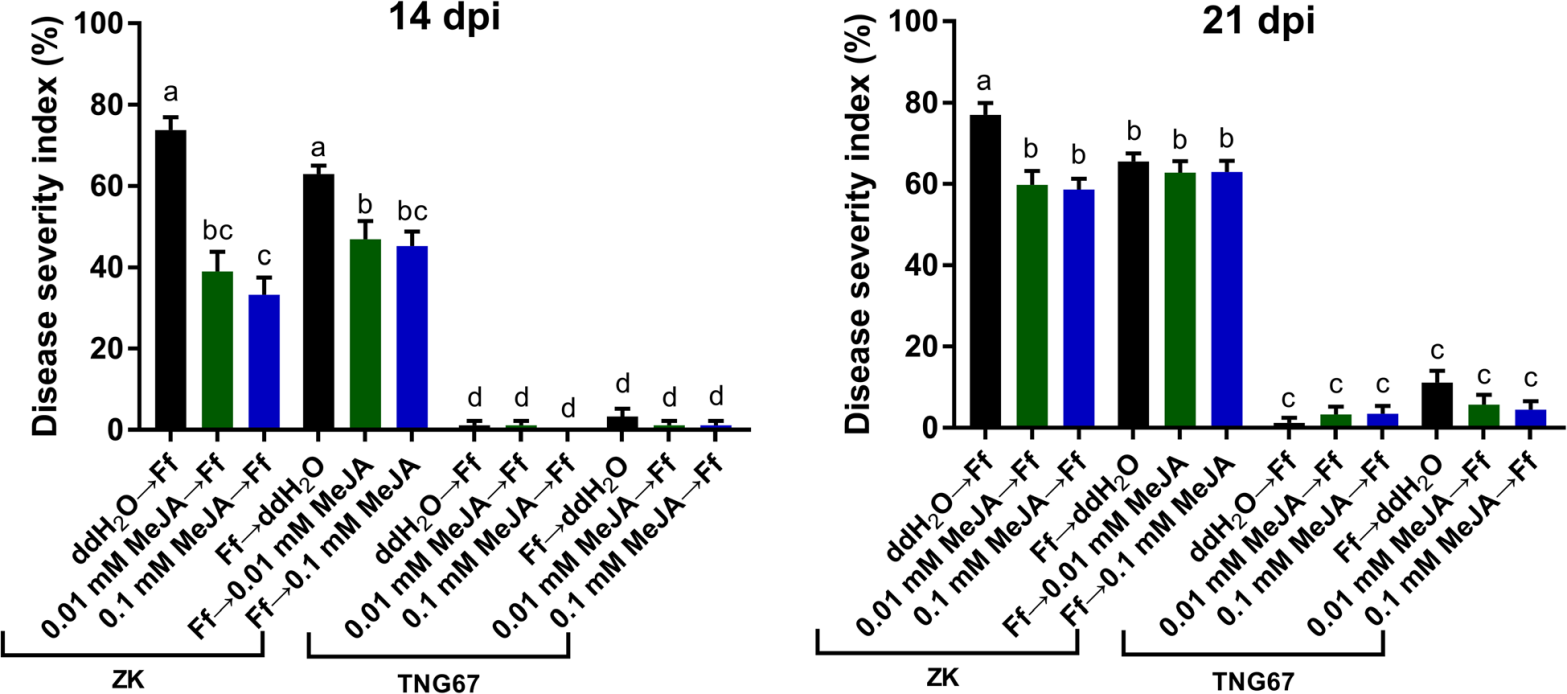
Effect of methyl jasmonate (MeJA) on disease severity indexes of Zerawchanica karatals (ZK) and Tainung 67 (TNG67) at 14 and 21 days post inoculation (dpi). Rice seeds were treated with ddH_2_O, 0.01 mM MeJA, or 0.1 mM MeJA before or after the inoculation of *Fusarium fujikuroi* (Ff). Data are mean ± SEM (*n* = 2 independent trials with 12–15 plants per treatment per trial). Different letters indicate significant difference based on Tukey’s multiple comparison test at *p* < 0.05

To understand whether JA mediates resistance to *F. fujikuroi* in rice, the effects of MeJA treatment (before or after *F. fujikuroi* inoculation) on disease severity at 14 dpi and 21 dpi were investigated (Fig. 6). For susceptible ZK, as compared with ddH_2_O treatment, MeJA treatment of rice seeds significantly alleviated and delayed the development of bakanae symptoms. No significant difference was observed between the 0.01 mM and 0.1 mM MeJA treatments; however, MeJA treatment before *F. fujikuroi* inoculation more effectively enhanced resistance than MeJA treatment after the inoculation. When treated with MeJA before inoculation, ZK plants showed a ~ 0.5- and ~ 0.8-fold reduction in DSI compared with the control at 14 and 21 dpi, respectively. MeJA treatment after inoculation caused a ~ 0.7-fold reduction and no significant reduction compared with the control at 14 and 21 dpi, respectively. Under all treatments, TNG67 exhibited similar levels of high resistance at 14 and 21 dpi (average DSI = 0%–11.1%).

### Effect of MeJA on the Viability of *F. fujikuroi*

To ensure that the observed JA-induced bakanae resistance was not due to the inhibitory effect of JA on the pathogen, *F. fujikuroi* spores were treated with MeJA or ddH_2_O then evaluated for germination rate and colony growth. More than 99.5% of the spores germinated within 12 h, and the colony diameters were ~ 4 cm after 7 days and ~ 5.8 cm after 10 days of growth on 1/2 PDA (Additional file 7: Table S7). No significant differences were observed between the ddH_2_O, 0.01 mM MeJA, and 0.1 mM MeJA treatments.

## Discussion

While rice defenses against leaf pathogens such as *Magnaporthe oryzae* and *Xanthomonas oryzae* pv. *oryzae* have been extensively explored and elucidated (Azizi et al. 2016; Nasir et al. 2018; Liu et al. 2013; Ji et al. 2018b; White and Yang 2009), the modulation of immunity responses during interaction with the seed-borne pathogen *F. fujikuroi* remains largely unknown. Profiling of *F. fujikuroi*-induced gene expression in rice has previously involved analyses of rice leaves at 7 and 21 dpi (Ji et al. 2016; Matić et al. 2016). Although *F. fujikuroi* infection can cause abnormal growth of the whole rice seedling, our qPCR analysis revealed that *F. fujikuroi* colonizes the stem and roots, but not the leaves. Aiming to uncover the defense mechanisms at an early stage of pathogenesis, we sequenced the total RNA of *F. fujikuroi*-colonized rice stem tissues at 7 dpi at a higher coverage and with longer reads [in comparison with 87.2 million reads of 50 bp per read generated by Ji et al. 2016 and 17 million reads of 51 bp per read generated by Matić et al. 2016, we generated 40–62 million reads of 150 bp per read for each sample]. Our qRT-PCR analysis of 14 DEGs and two SA marker genes, using RNA samples isolated from individual plants from two additional inoculation trials, validated the expression pattern of up- and down-regulated defense-related genes.

In this study, DEGs associated with plant defense were mainly classified in three categories: PRRs, TFs, and JA signaling pathway-related genes. Fewer DEGs were found in resistant TNG67 (*n* = 118) than susceptible ZK (*n* = 169), which is consistent with the finding of Matić et al. (2016): the numbers of DEGs in resistant Selenio and susceptible Dorella were 80 and 1285 at 7 dpi, and 3119 and 5095 at 21 dpi, respectively (the criteria for calling DEGs were FDR≦0.05 and |fold change|≧2). The DEGs and enriched GO terms identified in this study were largely different from those identified in the previous two transcriptome studies (Ji et al. 2016; Matić et al. 2016), perhaps reflecting the difference between systemic resistance in leaves versus local defense in stem tissues. It also suggests complex and variable mechanisms governing resistance or susceptibility to *F. fujikuroi* infection in different cultivars. Notably, Ji et al. (2019) recently analyzed the proteomics of *F. fujikuroi*-infected plants at 7 dpi. The protein encoded by a DEG we identified in TNG67, *limonene synthase* (Os04g0340300; LOC4335518), was found to be up-regulated in both the resistant cultivar 93–11 and the susceptible cultivar Nipponbare by Ji et al. (2019). Limonene has antifungal and antibacterial activities; another limonene synthase gene *OsTPS19* in rice was found to be induced upon *M. oryzae* infection, and overexpression of *OsTPS19* enhanced resistance to *M. oryzae* (Chen et al. 2018).

In agreement with previous observations, we found that genes involved in phytohormone biosynthesis were not differentially expressed at 7 dpi. At 1 and 2 weeks post inoculation (wpi) with *F. fujikuroi*, similar quantities of JA, SA, GA, and ABA accumulated in inoculated and non-inoculated seedlings of both resistant and susceptible cultivars (Siciliano et al. 2015). At 3 wpi but not 1 wpi, genes associated with JA biosynthetic process were up-regulated in resistant Selenio and down-regulated in susceptible Dorella, and genes related to GA metabolic process were up-regulated in susceptible Dorella and down-regulated in resistant Selenio (Matić et al. 2016).

JA has been shown to mediate plant defense against wounding, insect herbivores, and necrotrophic, hemibiotrophic, and biotrophic pathogens (Zhang et al. 2017). Contrasting roles of the JA pathway in response to Fusarium pathogens have been reported. In Arabidopsis, JA signaling has been negatively and positively associated with resistance against *Fusarium graminearum* (Makandar et al. 2010), and this pathway can be hijacked by *Fusarium oxysporum* to promote disease development (Thatcher et al. 2009). In the tomato - *F. oxysporum* f. sp. *lycopersici* (Thaler et al. 2004), date palm - *F. oxysporum* f. sp. *albedinis* (Jaiti et al. 2009), banana - *F. oxysporum* f. sp. *cubense* (Sun et al. 2013), and cotton - *F. oxysporum* f. sp. *vasinfectum* (Konan et al. 2014) pathosystems, exogenous JA treatment induced host resistance by enhancing production of defense-related phytoalexins (Konan et al. 2014) and enzymes [e.g., polyphenoloxidase and peroxidase (Jaiti et al. 2009)]. In this study, while no differential expression of genes in SA- and GA-related signaling pathways was observed, we detected a significant decrease in *OsJAZ9*, *OsJAZ10*, and *OsJAZ13* transcripts in both ZK and TNG67 at 3 and 7 days after *F. fujikuroi* inoculation. Because *JAZ* is a key repressor of JA signaling, the results suggested the activation of JA signaling upon *F. fujikuroi* infection. We did not observe differential expression of JA- or SA-regulated downstream defense genes (e.g., *OsPR1a*, *OsPR1b*, *OsWRKY45*, *OsJAmyb*) (Agrawal et al. 2001; Shimono et al. 2007; Agrawal et al. 2000; Lee et al. 2001) in the transcriptome data, perhaps because the stem samples included both *F. fujikuroi*-colonized and non-colonized tissues, and 7 dpi may be too early to detect systemic induction of the whole defense network.

JA signaling appears to play a crucial role in mediating early-stage defense responses against *F. fujikuroi* in rice. Exogenous JA treatment of rice seeds, prior to or after *F. fujikuroi* inoculation, provided enhanced resistance as evidenced by alleviated bakanae symptoms in susceptible ZK. Previous studies showed that exogenous application of MeJA to rice plants induced resistance to the rice blast pathogen *M. oryzae* (Han and Kahmann 2019), the root knot nematode *Meloidogyne graminicola* (Nahar et al. 2011; Kyndt et al. 2017), and Rice ragged stunt virus (RRSV) (Zhang et al. 2016). During initial stage of *M. oryze* infection, a fungal secreted monooxygenase may be employed to convert endogenous free JA to 12OH-JA, thus preventing the induction of rice immunity by fungal and host-derived JA (Patkar et al. 2015). RRSV infection in rice was found to induce the production of miR319, which suppressed JA-mediated defense and promoted disease development (Zhang et al. 2016). *F. fujikuroi* behaves like a necrotroph in susceptible rice genotypes (Ma et al. 2013; Matić et al. 2016), and effectors from necrotrophic pathogens were found to activate the SA pathway while suppressing the JA pathway (Tanaka et al. 2015) [e.g., the exopolysaccharide effector from *Botrytis cinerea* in tomato (El Oirdi et al. 2011)]. In this study, *OsNPR1* was up-regulated in susceptible ZK but not in resistant TNG67 at 7 dpi, suggesting the modulation of antagonistic crosstalk between the SA and JA pathways in ZK. GAs produced by *F. fujikuroi* and the host plant could also participate in the regulation of JA signaling through the degradation of DELLA proteins (the repressors of JAZs) (Navarro et al. 2008; De Vleesschauwer et al. 2016). It will be intriguing to elucidate how *F. fujikuroi* manipulates the phytohormone balance in susceptible rice cultivars to its benefit.

PRRs, WRKYs, and ERFs have been reported to be involved in signal perception and transduction pathways in plant defense and development (Macho and Zipfel 2014; Phukan et al. 2016; Müller and Munné-Bosch 2015). In this study, we identified four *PRRs* (*Os03g0297800*, *XIAO*, *Os08g0117700*, and *Os09g0110100*), four *WRKYs* (*OsWRKY21*, *OsWRKY24*, *OsWRKY28*, and *OsWRKY71*), and three *ERFs* (*OsERF53*, *OsERF54*, and *OsERF133*) that exhibited consistent differential expression patterns in both transcriptome and qRT-PCR analyses (contradictory results were only observed for the PRR gene *Os08g0117700* in TNG67 and *OsERF53* in ZK). Except for *OsWRKY28* and *OsWRKY71*, these selected DEGs were shown for the first time to be associated with disease resistance. Among the four *PRRs* encoding LRR receptor-like kinases, *XIAO* was previously found to participate in the regulation of brassinosteroid signaling and cell cycling in rice (Jiang et al. 2012). The *xiao* mutant displays dwarfism, smaller leaves, flower organs and seeds, and erect leaves (Jiang et al. 2012). At 7 dpi of *F. fujikuroi*, *XIAO* was up-regulated in resistant TNG67 and down-regulated in susceptible ZK, indicating a role of brassinosteroids in modulating rice responses to *F. fujikuroi*.

The four *OsWRKY* genes we identified were significantly down-regulated in ZK (*WRKY21* and *WRKY24*) or TNG67 (*WRKY71* and *WRKY28*) at 7 dpi. In Nipponbare, *OsWRKY28* is a negative regulator of basal defense responses against *M. oryzae* (Chujo et al. 2013). *OsWRKY71* was annotated as a TF involved in plant defense response (Liu et al. 2007). *OsWRKY71* was induced as early as 0.5 h after treatment with SA, MeJA, or 1-aminocyclo-propane-1-carboxylic acid (ACC; the precursor of ethylene), wounding, or *X. oryzae* pv. *oryzae* infection, and overexpression of *OsWRKY71* activated *OsNPR1* and *OsPR1b* (Liu et al. 2007). Notably, Ji et al. (2016) showed that *OsWRKY71* was up-regulated at 7 dpi in the moderately resistant cultivar 93–11. The opposite expression patterns of *OsWRKY71* in 93–11 and TNG67 implies different regulation of *OsWRKY71* in the two resistant cultivars. The three *ERF* genes we identified were significantly up- or down-regulated in ZK or TNG67 at 7 dpi, suggesting that they function differently in regulating rice resistance to *F. fujikuroi*. Contrasting roles in disease resistance have been observed for different *ERF* genes. For example, *OsERF922* and *OsERF83* were found negatively and positively regulate rice resistance to *M. oryzae* in rice cultivars Zhonghua 17 and Yukihikari, respectively (Liu et al. 2012; Tezuka et al. 2019).

## Conclusions

This study focused on investigating the early lines of defense against *F. fujikuroi* in rice seedlings. By comparing the transcriptomes of *F. fujikuroi*-infected and healthy stem tissues from plants at 7 dpi, we identified 169 DEGs in a susceptible cultivar, ZK, and 118 DEGs in a resistant cultivar, TNG67. qRT-PCR analysis on an individual plant basis enabled precise quantification and validation of 14 DEGs, most of which [12 DEGs, i.e., *Os03g0297800*, *XIAO*, *Os08g0117700*, *Os09g0110100*, *OsWRKY21*, *OsWRKY24*, *OsERF53*, *OsERF54*, *OsERF133*, *OsJAZ9*, *OsJAZ10*, and *OsJAZ13*] had never been associated with disease resistance. Interestingly, among complex phytohormone biosynthesis and signaling pathways, only JA signaling pathway genes were identified as DEGs. Significant repression of *OsJAZ9*, *OsJAZ10*, and *OsJAZ13* at 3 dpi and 7 dpi in both cultivars, indicated the activation of JA signaling during early interactions between rice and *F. fujikuroi*. Exogenous MeJA treatment of rice seeds could delay bakanae disease development in susceptible ZK, which also demonstrates the pivotal role of JA in rice resistance against *F. fujikuroi*. Detailed mechanisms underlying JA-mediated bakanae resistance and the novel defense-related DEGs are worthy of further investigation. Moreover, to have comprehensive knowledge on rice defenses in response to *F. fujikuroi* colonization, root transcriptome profiles also deserve to be explored.

## Supplementary information

**Additional file 1: Table S1.** Primers used for real-time quantitative reverse transcription PCR (qRT-PCR)

**Additional file 2: Table S2.** Mapping results for RNA-seq reads from dH_2_O-treated or *F. fujikuroi*-inoculated Zerawchanica karatals (ZK) and Tainung 67 (TNG67)

**Additional file 3: Table S3.** Expression levels and functional annotations for all Zerawchanica karatals (ZK) and Tainung 67 (TNG67) transcripts

**Additional file 4: Table S4.** List of differentially expressed genes (DEGs) in Zerawchanica karatals (ZK) and Tainung 67 (TNG67)

**Additional file 5: Table S5.** List of genes involved in jasmonic acid, ethylene, salicylic acid and gibberellic acid biosynthesis or signaling pathways

**Additional file 6: Table S6.** Expression profiling of 14 selected genes by RNA-seq and real-time quantitative RT-PCR (qRT-PCR)

**Additional file 7: Table S7.** Effects of methyl jasmonate (MeJA) on the germination rate and colony growth of *Fusarium fujikuroi*

**Additional file 8: Fig. S1.** Distribution of bakanae disease severity indexes of 231 accessions in rice diversity panel 1. The resistance scores of Zerawchanica karatals (ZK) and Tainung 67 (TNG67) are indicated by arrows. Data from the study of Chen et al. (2019) were adjusted using best linear unbiased estimates (BLUEs) in TASSEL 5.2.24 for control of variation among blocks in different inoculation trials.

**Additional file 9: Fig. S2.** Symptoms of Zerawchanica karatals (ZK) and Tainung 67 (TNG67) after dH_2_O (mock) or *Fusarium fujikuroi* (Ff) inoculation at 21 days post inoculation

**Additional file 10: Fig. S3.** *Fusarium fujikuroi* genomic DNA standard curves for quantitative real-time PCR (qPCR). A dilution series of *F. fujikuroi* DNA was mixed with DNA from different rice tissues collected from healthy Zerawchanica karatals (ZK) or Tainung 67 (TNG67) seedlings. (a) 3 days post dH_2_O treatment; (b) 7 days post dH_2_O treatment. The linear regression equations and their coefficient of determination (*R*^*2*^) values are shown on the graph.

**Additional file 11: Fig. S4.** Transcriptome profiles in *Fusarium fujikuroi*-inoculated Zerawchanica karatals (ZK) and Tainung 67 (TNG67). (a) Principal component analysis (PCA) for the RNA-seq samples. Mock: dH_2_O-treated; Ff: *F. fujikuroi*-inoculated; 1: pooled sample from independent trial 1; 2: pooled sample from independent trial 2. (b) Numbers of differentially expressed genes (DEGs) in the two cultivars. (c) Heat map of the expression levels (log2 fold change) of DEGs. Red represents up-regulation and blue represents down-regulation in *Fusarium fujikuroi*-inoculated plants.

## Data Availability

RNA-seq data were deposited in the NCBI Sequence Read Archive database (SAMN13972374 to SAMN13972381). Other datasets used and analyzed during the current study are available from the corresponding author on reasonable request.

## Abbreviations

ABA: Abscisic acid
ACC: 1-aminocyclo-propane-1-carboxylic acid
ANOVA: Analysis of variance
Ct: Cycle threshold
CTAB: Cetyltrimethylammonium bromide
DAMPs: Damage-associated molecular patterns
DEGs: Differentially expressed genes
DPI: Days post inoculation
DSI: Disease severity index
EF1α: Elongation factor 1-alpha
ERFs: Ethylene response factors
FDR: False discovery rate
GA: Gibberellin
GO: Gene ontology
HDA: Histone deacetylase
JA: Jasmonic acid
JAZ: Jasmonate ZIM-domain
MAP3K: Mitogen-activated protein kinase kinase kinase
MeJA: Methyl jasmonate
NPR: Non-expressor of pathogenesis-related gene
PAL: Phenylalanine ammonia-lyase gene
PAMPs: Pathogen-associated molecular patterns
PDA: Potato dextrose agar
POEI: Pollen Ole e I
PRRs: Pattern-recognition receptors
PTI: Pattern-triggered immunity
qPCR: Real-time quantitative PCR
qRT-PCR: Real-time quantitative reverse transcription PCR
QTL: Quantitative trait locus
RDP1: Rice diversity panel 1
RRSV: Rice ragged stunt virus
SA: Salicylic acid
TFs: Transcription factors
WAK: Wall-associated kinase
WPI: Weeks post inoculation
