# Supplementary material for: Transcriptome Analysis of Early Defenses in Rice against Fusarium fujikuroi
Source: Rice (N Y). 2020 Sep 10;13:65. doi: 10.1186/s12284-020-00426-z (PMC7483690; doi:10.1186/s12284-020-00426-z)
Supplement: Supplementary file 7 — Additional file 7: Table S7. Effects of methyl jasmonate (MeJA) on the germination rate and colony growth of Fusarium fujikuroi [file 12284_2020_426_MOESM7_ESM.docx]

**Additional file 7: Table S7. Effects of methyl jasmonate (MeJA) on the germination rate and colony growth of *Fusarium fujikuroi***

| **Treatment** | **Spore germination rate (%)^a^** | **Colony diameter (cm)^a^** | |
| --- | --- | --- | --- |
|  |  | **7 days** | **10 days** |
| **ddH_2_O** | 99.67 ± 0.21 a | 4.08 ± 0.02 a | 5.85 ± 0.03 a |
| **0.01 mM MeJA** | 99.50 ± 0.34 a | 4.05 ± 0.03 a | 5.81 ± 0.03 a |
| **0.1 mM MeJA** | 99.83 ± 0.17 a | 4.01 ± 0.03 a | 5.79 ± 0.03 a |

^a^Data are mean ± SEM. Same letters indicate no significant difference based on Tukey's multiple comparison test at *p* < 0.05.
