# Supplementary figures and images for: Transcriptome Analysis of Early Defenses in Rice against Fusarium fujikuroi
Source: Rice (N Y). 2020 Sep 10;13:65. doi: 10.1186/s12284-020-00426-z (PMC7483690; doi:10.1186/s12284-020-00426-z)

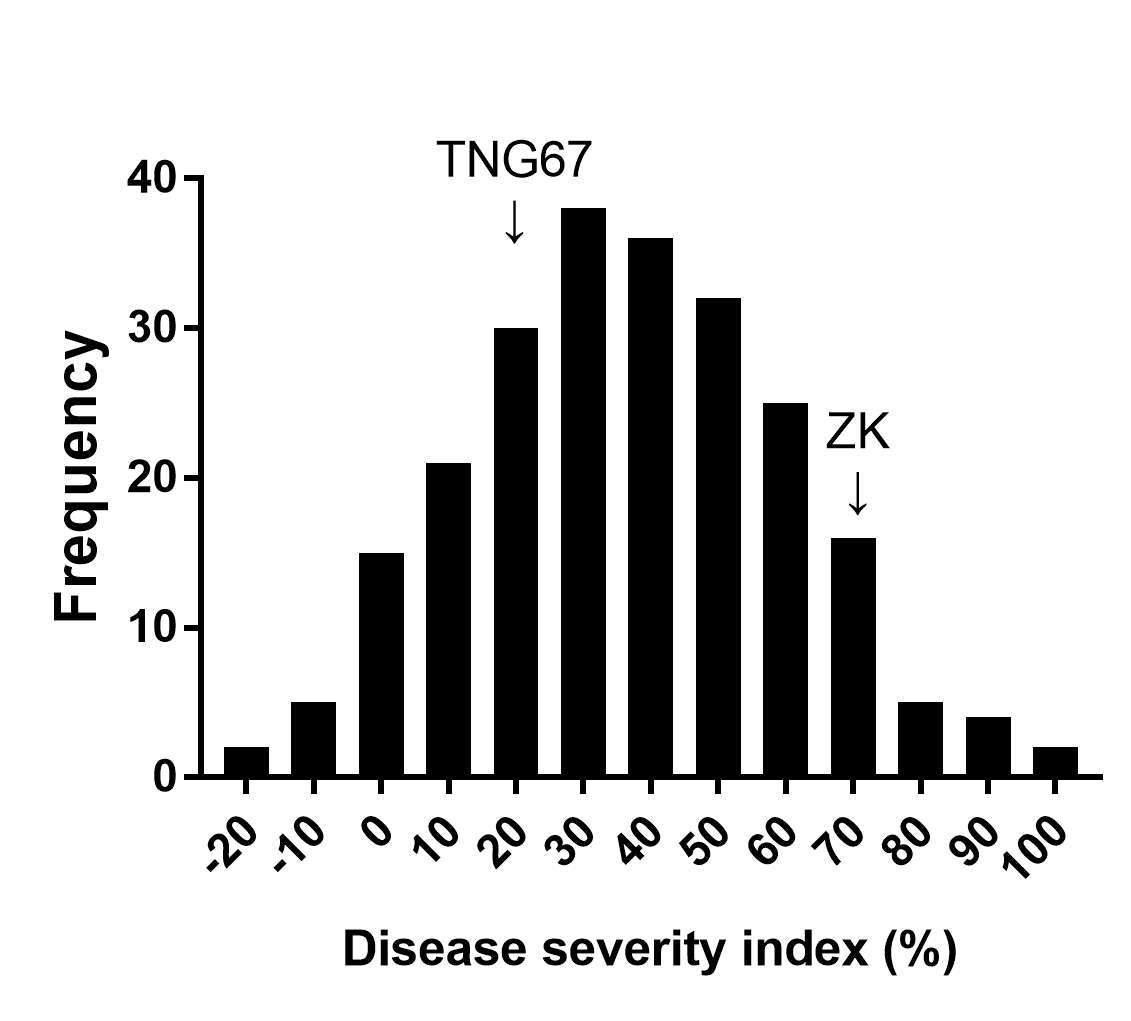

Supplement: Supplementary file 8 — Additional file 8: Fig. S1. Distribution of bakanae disease severity indexes of 231 accessions in rice diversity panel 1. The resistance scores of Zerawchanica karatals (ZK) and Tainung 67 (TNG67) are indicated by arrows. Data from the study of Chen et al. (2019) were adjusted using best linear unbiased estimates (BLUEs) in TASSEL 5.2.24 for control of variation among blocks in different inoculation trials. [file 12284_2020_426_MOESM8_ESM.tif]

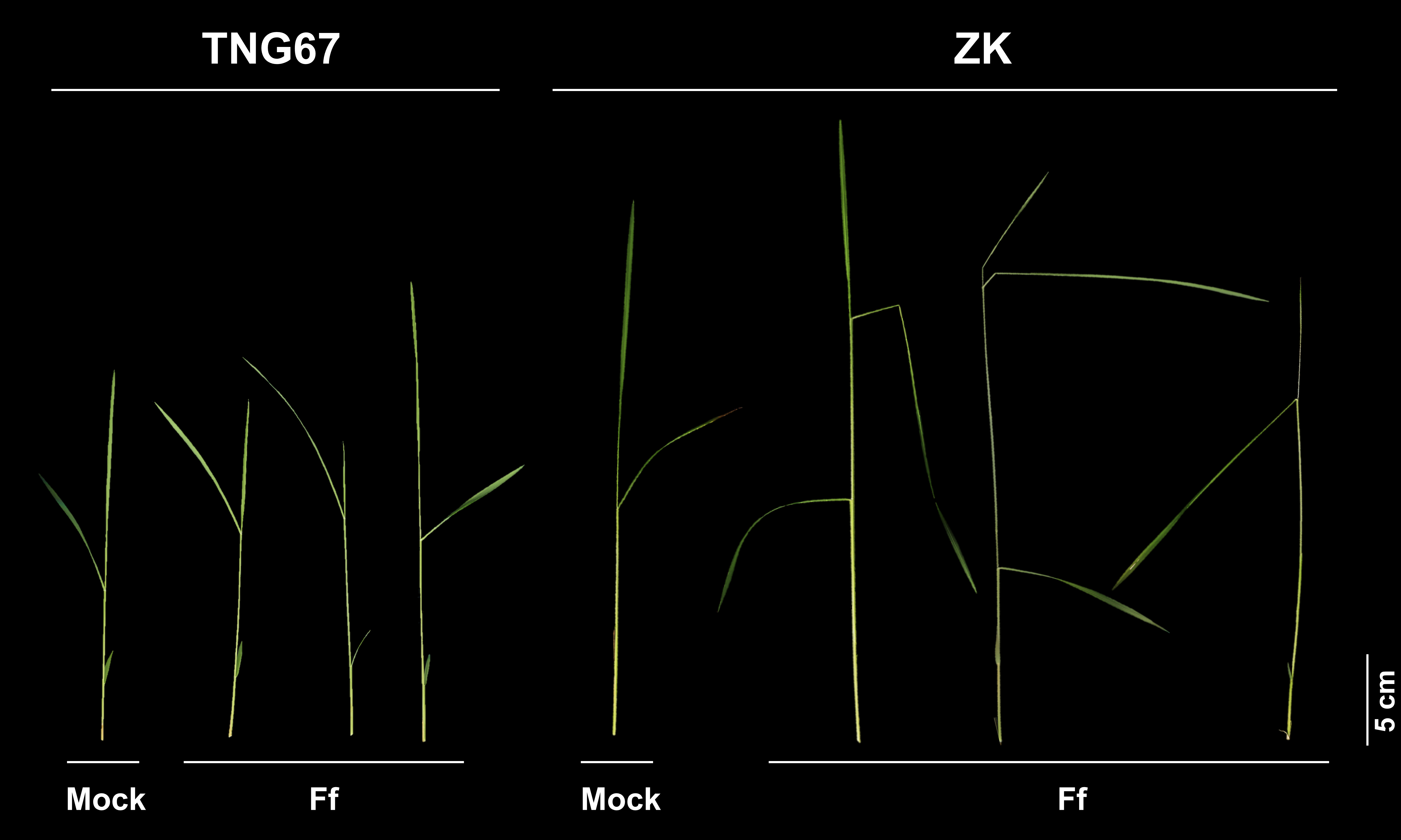

Supplement: Supplementary file 9 — Additional file 9: Fig. S2. Symptoms of Zerawchanica karatals (ZK) and Tainung 67 (TNG67) after dH2O (mock) or Fusarium fujikuroi (Ff) inoculation at 21 days post inoculation [file 12284_2020_426_MOESM9_ESM.tif]

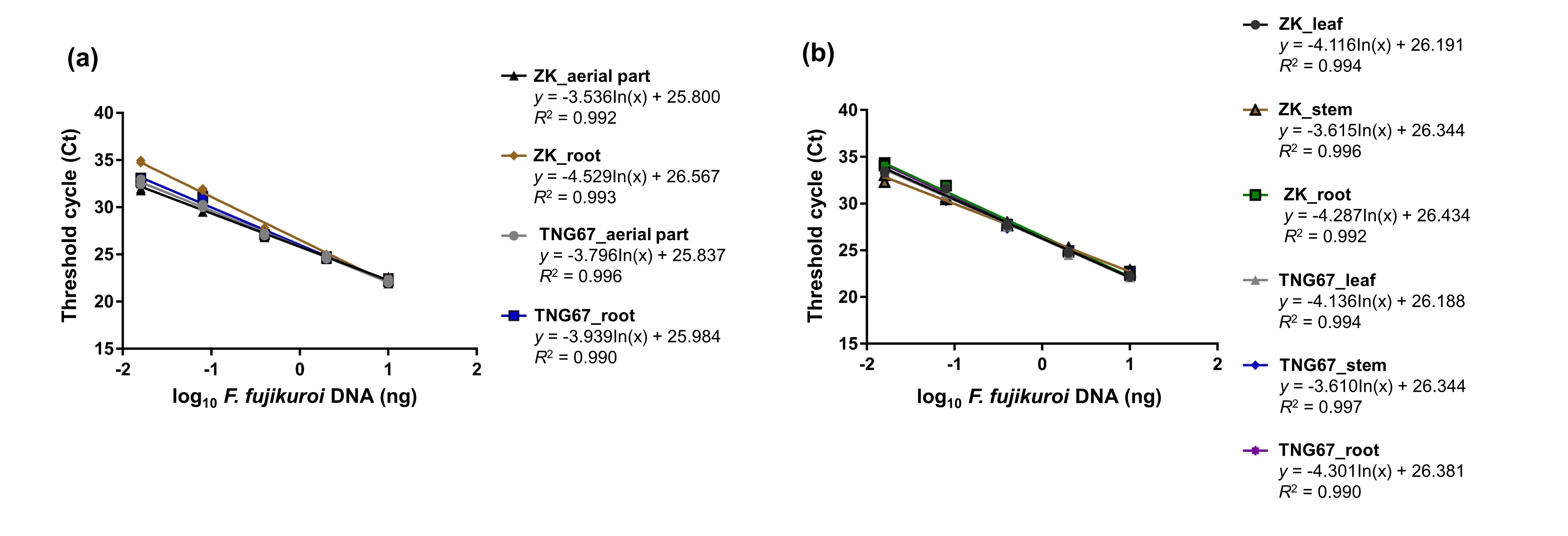

Supplement: Supplementary file 10 — Additional file 10: Fig. S3. Fusarium fujikuroi genomic DNA standard curves for quantitative real-time PCR (qPCR). A dilution series of F. fujikuroi DNA was mixed with DNA from different rice tissues collected from healthy Zerawchanica karatals (ZK) or Tainung 67 (TNG67) seedlings. (a) 3 days post dH2O treatment; (b) 7 days post dH2O treatment. The linear regression equations and their coefficient of determination (R2) values are shown on the graph. [file 12284_2020_426_MOESM10_ESM.jpg]

**a**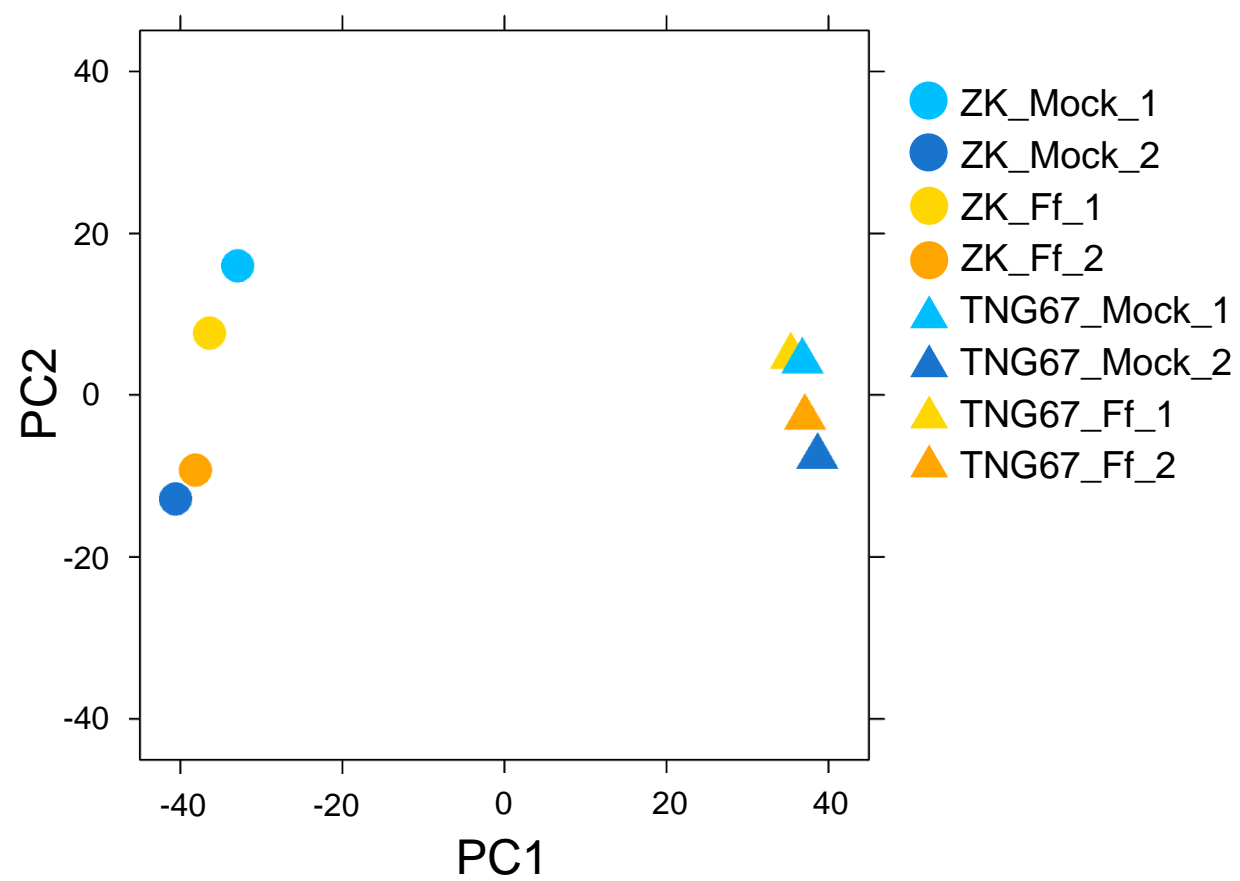**b**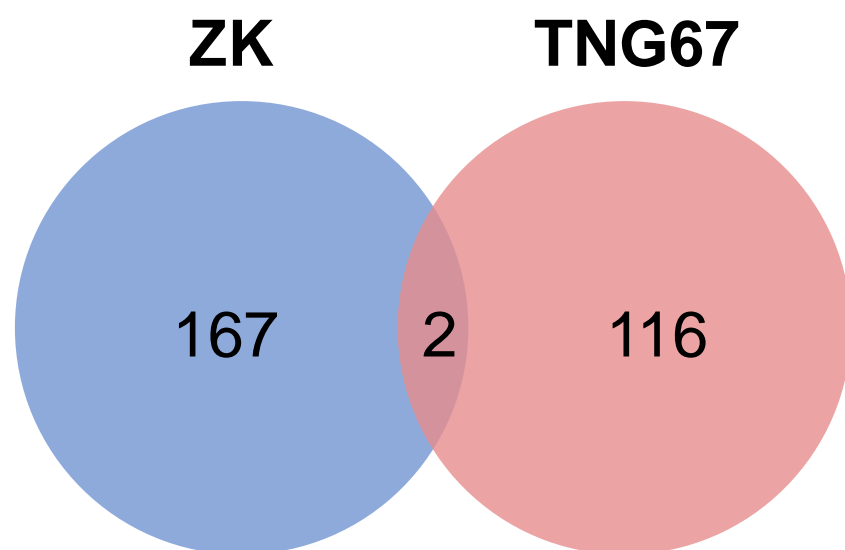**c**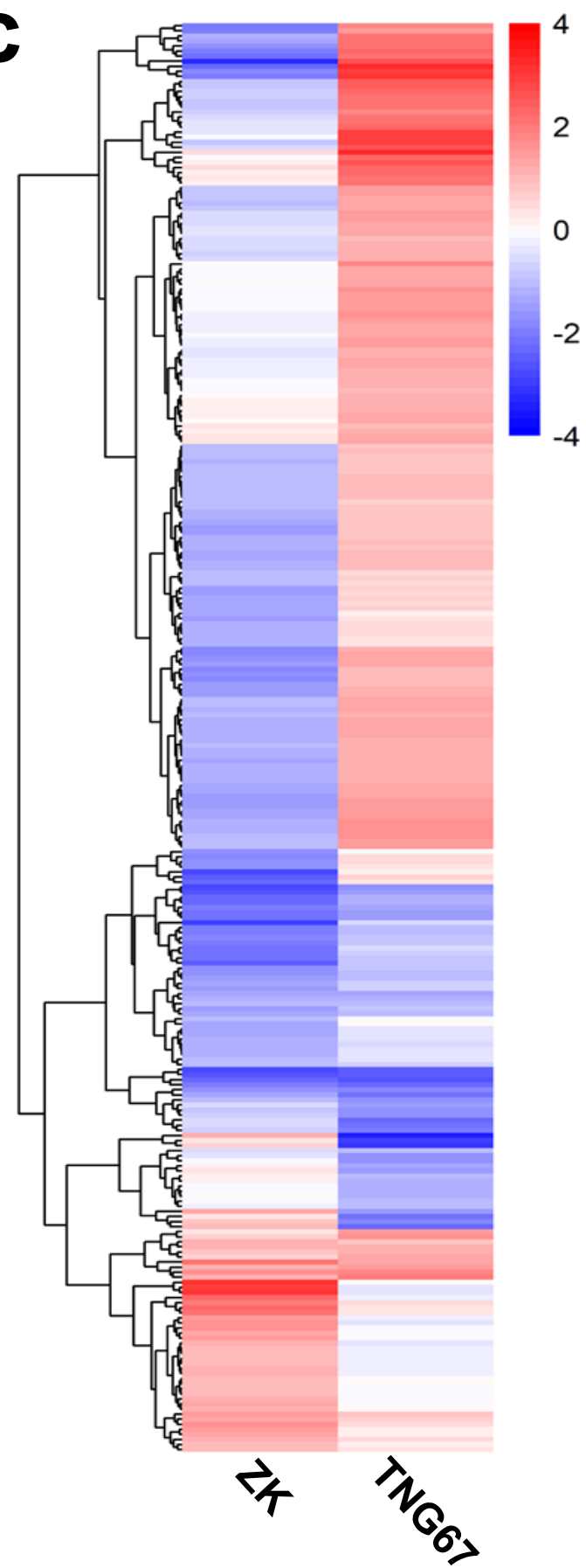

Supplement: Supplementary file 11 — Additional file 11: Fig. S4. Transcriptome profiles in Fusarium fujikuroi-inoculated Zerawchanica karatals (ZK) and Tainung 67 (TNG67). (a) Principal component analysis (PCA) for the RNA-seq samples. Mock: dH2O-treated; Ff: F. fujikuroi-inoculated; 1: pooled sample from independent trial 1; 2: pooled sample from independent trial 2. (b) Numbers of differentially expressed genes (DEGs) in the two cultivars. (c) Heat map of the expression levels (log2 fold change) of DEGs. Red represents up-regulation and blue represents down-regulation in Fusarium fujikuroi-inoculated plants. [file 12284_2020_426_MOESM11_ESM.pdf]
